# Suitability of accelerometry as an objective measure for upper extremity use in stroke patients

**DOI:** 10.1186/s12883-022-02743-w

**Published:** 2022-06-15

**Authors:** Anne-Lisa Heye, Christine Kersting, Malte Kneer, Anne Barzel

**Affiliations:** 1 Chair of General Practice I and Interprofessional Care, Institute of General Practice and Primary Care, Faculty of Health, Witten/Herdecke University, Alfred-Herrhausen-Str. 50, 58448 Witten, Germany; 2 Chair of General Practice II and Patient-Centeredness in Primary Care, Institute of General Practice and Primary Care, Faculty of Health, Witten/Herdecke University, Alfred-Herrhausen-Str. 50, 58448 Witten, Germany; 3 Institute of General Medicine, Ulm University, Albert-Einstein-Allee 23, 89081 Ulm, Germany

**Keywords:** Accelerometry, Stroke, Activities of daily living, Paresis, Upper limb

## Abstract

**Background:**

Upper limb (UL) paresis is one of the most common stroke consequences and significantly restricts patients in everyday life. Instruments objectively measuring direct arm use in stroke patients are lacking, but might be helpful to understand patients’ impairment. Aiming to examine whether accelerometry is a suitable objective measure for everyday UL use in stroke patients, we conducted a systematic review on the association between accelerometer-derived measurements and clinical scales.

**Methods:**

Articles were systematically searched in PubMed, Scopus, Cochrane Library, PeDro and LIVIVO through December 12^th^, 2021, screened for inclusion by AH, and subsequently independently screened by CK and MK. Disagreements were discussed until consensus. We included English and German peer-reviewed articles dealing with the validity of accelerometers as a measurement of UL use in stroke patients and eligible systematic reviews. Studies exclusively using accelerometry as an outcome parameter, book contributions, conference abstracts and case studies were excluded. Data extraction was conducted by AH and confirmed by CK focussing on study type, objective, accelerometer device, sample size, stroke status, assessments conducted, measurement method, wearing time and key results. We analysed all eligible articles regarding the correlation between accelerometry and other clinical assessments and the validity in accordance with the type of accelerometer.

**Results:**

Excluding duplicates, the initial search yielded 477 records. In the 34 eligible studies accelerometers was used with a predominance of tri-axial accelerometery (*n* = 12) and only few with two-axial application (*n* = 4). Regarding measures to examine association to accelerometer data different clinical scales were applied depending on the setting, the degree of impairment and/or the status of stroke. Cut-off values to determine correlations varied largely; most significant correlations are reported for the MAL [Range 0.31- 0.84] and the ARAT [Range 0.15–0.79].

**Conclusions:**

Accelerometers can provide reliable data about daily arm use frequency but do not supply information about the movements´ quality and restrictions on everyday activities of stroke patients. Depending on the context, it is advisable to use both, accelerometry and other clinical measures. According to the literature there is currently no accelerometer device most suitable to measure UL activity. High correlations indicate that multi-dimensional accelerometers should be preferred.

**Supplementary Information:**

The online version contains supplementary material available at 10.1186/s12883-022-02743-w.

## Background

Arm impairment is one of the most common consequences of a stroke, resulting in significant long-term impairment, and severely restricts patients in their everyday life [1, 2]. Hartman-Maeir et al. [3] estimate, that stroke patients with arm impairment discontinue up to 57% of meaningful activities [4]. Accordingly, to enhance the autonomy of stroke patients, restoring arm use is a major treatment goal [5]. Studies indicate that daily use of the affected arm positively influences upper extremity (UE) function [6, 7] as well as patients’ quality of life [8]. In order to assess the severity of the impairment and in particular arm function, reliable instruments are required to allow reasonable therapy planning as well as an evaluation of therapy success [9]. Indeed, daily activities of patients may not be adequately reflected in inpatient settings and hospitals [10], which calls for measuring UL use outside the clinic [11, 12].

There are different approaches to assess UL use after stroke, including patients’ self-report (e.g., Stroke Impact Scale, Motor Activity Log) [13–15], therapists’ observation, and/or timekeeping [5]. In general, clinical tests tend to focus on UL function rather than on everyday life use. However, improved function does not necessarily go hand-in-hand with increased UL use in everyday activities at home [16, 17] indicating that UL functional capacity should be assessed separately from the actual UL use [4, 11, 12, 18]. This necessity is underlined by the phenomenon of so-called “learned non-use”, meaning that stroke patients learn to more frequently use their unimpaired instead of their impaired arm for daily activities, which might thereby become a habit even when the function of the impaired arm recovers [19, 20].

Measurements established in post-stroke rehabilitation therapy of UL focus on different areas of impairment. Whereas some address the degree of impairment (e.g., grip strength, Fugl-Meyer Assessment, FMA), others assess activity (e.g., Action Research Arm Test, ARAT, WOLF Motor Function Test, WMFT) or participation (e.g., Motor Activity Log, MAL) [5]. Performing assessments is often time-consuming and may be limited due to the need for specific test material and/or intensive training of the therapist [20]. Furthermore, many of the commonly used tests require particular patient skills or may only be applicable to a selected patient population. Thus, a standardised procedure may not always be suitable. Moreover, taking a look at self-reported measures, there is a need for reliable, objective and reproducible assessments in rehabilitation to minimise subjective bias [21], such as report bias due to cognitive impairment and social desirability [12]. In a systematic review on valid and reliable instruments for arm-hand assessments in persons with hemiplegia, Lemmens et al. (2012) identified only two available instruments with regard to level of arm use: the Motor Activity Log and accelerometry [22]. While the MAL is predominantly driven as a (self-) assessment of the quality and the amount of arm/hand use with regard to prespecified activities of daily living, accelerometry [23–25] can be used in stroke patients to quantify arm use at different phases during the rehabilitation process [10, 26] and different levels of impairment [10, 27]. However, so far, no gold standard for measuring UL use post-stroke has been established. Accordingly, guidelines rarely give recommendations as to which measurement should be used [5, 28].

Although accelerometry allows for an objective and reliable measurement of direct arm use in patients with UL paresis after stroke, accelerometry has not yet been established as the gold standard. Evidence supporting the use of accelerometers is increasing, but the available data for UL use in daily life are limited, particularly with regard to outpatient stroke rehabilitation [4, 23].

Against this background, we conceptualised a validation study to assess whether wrist-worn accelerometry, in particular tri-axial accelerometers, is suitable as an objective measure for everyday UL use in stroke patients with UL motor impairment. To prepare this study we conducted a systematic review of the international literature to provide an overview of the available evidence regarding the association between accelerometer-derived measurements and commonly used clinical scales to map everyday UL function.

## Methods

We conducted a systematic literature search in PubMed (including Medline), Cochrane Library, Scopus, PeDro and LIVIVO. We did not limit the search period. The search was last updated on December 12^th^, 2021. Results are reported in accordance with the PRISMA guidelines [29].

### Search strategies

The first search was created on the basis of the PICO framework (Population, Intervention, Comparison, Outcome) and conducted in PubMed using MESH terms and keywords (Additional file 1). The search algorithm was then adapted for the other databases. Articles had to include the following terms: (1) accelerometry or actigraphy, (2) stroke or apoplexy, (3) upper extremity, arm activity or physical activity, (4) paresis or motor impairment. Keywords for which no MeSH term exists were searched in all fields. Boolean operators were chosen to specify matches by excluding studies that examine the use of accelerometry in other contexts. Used terms correspond to keywords of known eligible articles and of used search algorithms in eligible systematic reviews. The search term was: ((((((((((((Accelerometr*) OR Accelerometry[MeSH Terms]) OR Actigraph*)) AND (((stroke) OR stroke[MeSH Terms]) OR apoplexy))) AND (((Upper extremity) OR Arm activity) OR physical activity))) AND ((paresis) OR Motor impairment))))). There were no limitations. Hits were exported to EndNote X8.

### Eligibility criteria

We considered articles as eligible when they were peer-reviewed, written in either German or English, and reported about the results of studies (systematic reviews, observational studies, validation studies, cross-sectional studies and prospective studies) evaluating the validity or reliability of accelerometry to measure daily use of UL in stroke patients with impaired arm function, independent of individuals’ stroke status (acute, subacute, chronic). We also screened and included articles with other objectives when UL use was monitored and correlations between accelerometry and different measures were reported. To allow a reasonable comparison we included only studies that used bilateral wrist-worn accelerometry. We excluded studies that validated complementary systems like the Stroke Upper-Limb Activity Monitor (ULAM) or the inertial measurement unit (IMU) system. Studies conducted among children and those solely applying accelerometry to measure the effect of an intervention were excluded. Articles without full-text availability were likewise excluded.

### Study selection

**Fig. 1:**
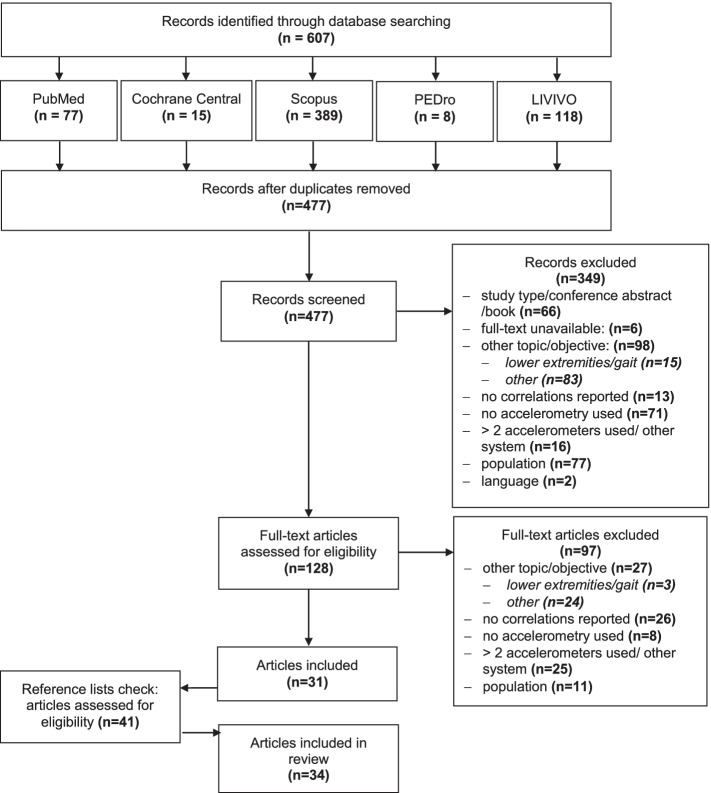
PRISMA flow diagram of the systematic review selection process based on PRISMA 2009 guidelines [29]

After removing duplicates, AH screened all remaining references on the basis of the title, abstract and potential eligible full texts. Two reviewers (CK, MK) independently screened references and full texts for inclusion. In the second step, we compared the articles that were considered eligible. Any disagreements were discussed until a consensus was achieved. Applying the "pyramid scheme" and snowballing technique, we additionally conducted a reference check of eligible articles and performed a manual search. Figure 1 illustrates the selection process in a PRISMA flow diagram.

#### Data collection process

In line with the objective of this review, we analysed all eligible articles regarding correlations between accelerometry and clinical assessments, and/or the validity in accordance with the type of accelerometer.

Data extraction and thematic analysis were used to synthesise the relevant information as well as to identity differences and enable a reasonable comparison of included studies. To analyse correlations, we extracted all data regarding study type, aim of the study, accelerometer device, sample size of participants, status of stroke disease (acute, subacute, chronic), assessments used for comparison, accelerometer measures (method, algorithm), wearing time and results. We then extracted data regarding the method of accelerometry measures (used epoch), level of UL impairment, setting and correlation coefficients considered. Extracted data were analysed to identify differences between studies regarding methodology and results. This systematic review describes the reported correlations for accelerometry recordings and different measures.

## Results

### Study selection

Excluding duplicates, the literature search yielded 477 references, of which 31 were included in this review. In addition, 41 articles were identified via a reference check and manual search; of these, three articles fulfilled the inclusion criteria, resulting in a total of 34 articles eligible for data extraction and analysis. Figure 1 shows the results of including and excluding references in a PRISMA flow diagram.

### Study characteristics

The 34 articles included reported on RCTs [10, 30, 31] (*n* = 3), clinical trials [32, 33] (*n* = 2), validation studies [14, 24, 25, 34–36] (*n* = 6), observational studies [18, 37–40] (*n* = 5), cross-sectional studies [6, 12, 41–45] (*n* = 7), prospective studies [27, 46, 47] (*n* = 3), exploratory studies [48] (*n* = 1), reviews [5, 49] (*n* = 2) and systematic reviews (*n* = 5) [22, 23, 28, 50, 51].

The number of study participants ranged from 10 to 222, but the majority of articles reported a sample size below 100 [6, 10, 12, 14, 18, 24, 27, 30, 31, 33–38, 40–45, 48].

The stroke status of patients was reported in 24 of the 34 included studies: Eleven articles reported on chronic stroke patients [12, 14, 24, 30, 31, 34, 35, 42, 44, 45, 48], seven on patients with acute stroke [10, 18, 27, 33, 39, 43, 46] and three articles referred to (early) subacute stroke [25, 32, 37]. Three studies included participants at different stages of rehabilitation after stroke [6, 40, 41]. In most of the study participants, the severity of UL impairment was rated as mild to moderate.

For more details on study characteristics and relevant information extracted from the studies, see Additional file 2, Table S1 and Table S2.

### Accelerometer devices to measure everyday UL use in stroke patients

The studies used different types of accelerometer devices to measure physical activity, e.g., uni-axial and multi-axial accelerometers. The included articles reported mainly about the use of tri-axial accelerometry (*n* = 12) [4, 6, 12, 18, 31, 33, 37, 40, 41, 44, 45, 48]. The use of uni-axial accelerometry was reported in five articles [10, 27, 39, 43, 46], two-axial accelerometry was mentioned in four articles [14, 24, 25, 32], and five articles did not comment on the type of accelerometer [30, 34–36, 42]. The duration of data collection via accelerometer ranged from a minimum of 22 h [40], between 24 h [4, 10, 18, 30, 31, 33, 36, 37, 41, 43], 25–26 h [12, 44], and 48 h [27, 39, 46], up to a maximum of 72 h [6, 14, 24, 25, 32, 34, 35, 42, 45]. In two articles accelerometers were worn only while performing specific activities and tasks [39, 48], and in one study the duration of data collection was not reported [36]. None of the included articles provided a rationale for the chosen wearing time of accelerometers.

The place of application of accelerometry measures varied reasonably between articles, including the community setting [12, 25, 44], a medical centre [34], the hospital setting [27, 33, 43] or a specific rehabilitation hospital/clinic/centre [6, 18, 35, 37, 38, 40, 48] and the patient’s home or free living environments outside the laboratory [14, 24, 30–32, 35, 40, 45]. The articles did not consistently focus on the daily use of the UL in stroke patients outside the laboratory or clinic [18, 23, 37, 47].

### Criteria for objectivity of accelerometry concerning everyday UL use in stroke patients

With regard to validation procedures studies included reported statistical calculations of correlations between accelerometer recordings and common clinical assessments using Spearman or Pearson coefficients as standard reference to measure validity. The cut-off values defining weak, moderate and strong correlations differ between studies. While some studies describe the strength of correlation by using conventions from meta-analysis literature, i.e., 0.1 as weak or low, 0.3 as moderate and 0.5 as strong (according to Cohen 1988 [52]), others describe coefficients between 0 and 0.25 as low, 0.25 to 0.5 as moderate or fair, and a value between 0.5 and 0.75 as a good to excellent relationship (according to Portney & Watkins, 1993 [53]). Urbin et al. [40, 48] considers the strength of correlation coefficients as moderate at 0.30 to 0.59, and as strong at 0.60 or greater.

We found a large variety of measures with a total of 20 clinical scales (Table S2) used in the studies to assess correlation between accelerometer-derived measurements and clinical scales. Clinical tests that showed correlations are outlined in Table 1. Correlations reported by studies referred predominantly on the MAL and the ARAT.

**Table 1:** Correlations between accelerometry (use ratio) and clinical tests for upper extremity

Clinical test	Correlation reported by studies		Ref	Significance level
	**Pearson’s** **r value**	**Spearman`s p value**		
AAUT^a^	*r* = 0.60		[25]	*p* = 0.001
ABILHAND^b^	*r* = 0.45–0.54		[36]	
ARAT^c^		*ρ* = 0.40	[10]	
		*ρ* = 0.66	[12]	*p* < 0.001
	*r* = 0.65		[18]	
		*ρ* = -0.57	[31]	*p* < 0.01
		*ρ* = 0.79	[40]	*p* < 0.001
		*ρ* = 0.63	[44]	
		*ρ* = 0.50	[45]	*p* < 0.05
		*ρ* = 0.15–0.57	[48]	*ρ* > 0.38 = *p* < 0.05 *ρ* > 0.48 = *p* < 0.01
BRS^d^	*r* = 0.77		[41]	*p* < 0.05
FAABOS^e^	*r* = 0.55		[35]	*p* < 0.05
FIM^f^ Motor / UE function		*ρ* = 0.67 / *ρ* = 0.58	[10]	
FIM^f^	*r* = 0.50		[41]	*p* < 0.05
FMA^g /^ UEFM	*r* = 0.69		[18]	
		*ρ* = 0.54	[27]	*p* = 0.001
		*ρ* = 0.28–0.73	[39]	
		*ρ* = -0.85	[43]	*p* < 0.001
		*ρ* = 0.60	[45]	*p* < 0.001
		*ρ *= 0.70	[46]	
		*ρ* = 0.70	[47]	
MAL^h^ MAL-AOU/QOM		*ρ* = 0.60 / *ρ* = 0.66	[6]	*p* < 0.001
AOU/QOM	*r* = 0.91 / *r* = 0.73		[14]	*p* = 0.01
	*r* = 0.71		[24]	*p* = 0.001
	*r* = 0.52		[25]	*p* = 0.001
AOU/QOM	*r* = 0.47 / *r* = 0.52		[32]	*p* = 0.01
Pre/post-intervention	*r* = 0.31 / *r* = 0.52		[30]	*p* = 0.01 / *p* < 0.001
	*r* = 0.47		[34]	
AOU/QOM	*r* = 0.84 / *r* = 0.79		[41]	*p* < 0.05
		*ρ* = 0.61	[42]	*p* < 0.001
AOU/QOM		*ρ* = 0.58 /*ρ* = 0.61	[45]	*p* < 0.01
mRS^i^		*ρ* = -0.13 - -0.49	[39]	
		*ρ* = -0.48	[46]	
		*ρ* = -0.48	[47]	
NEADL^j^	*r* = 0.34		[34]	
NIHSS^k^	*r* = -0.69		[18]	*p* = 0.01
		*ρ* = –0.59	[27]	*p* = 0.001
		*ρ* = -0.25 - -0.49	[39]	
	*r* = -0.60		[41]	
		*ρ* = -0.51	[46]	
SIS^l^ Subscale Hand Function / Mobility		*ρ* = 0.58/ *ρ* = 0.23	[6]	*p* < 0.001/ *p* < 0.001
	*r* = 0.16		[25]	*p* = 0.05
	*r* = 0.42		[34]	
WMFT^m^ Function / Time		*ρ* = 0.62/ *ρ* = -0.65	[10]	
Function		*ρ* = 0.59	[45]	*p* < 0.01

^a^Actual Amount of Use Test

^b^Test of manual ability of the UE

^c^Action Research Arm Test

^d^Brunnstrom Recovery Stage

^e^Functional Arm Activity Behavioral Observation System

^f^Functional Independence Measure for motor and UE function

^g^Fugl-Meyer Assessment

^h^Motor Activity Log

^i^modified Rankin Scale

^j^Nottingham Extended Activities of Daily Living

^k^National Institute of Health Stroke Scale

^l^Stroke Impact Scale

^m^Wolf Motor Function Test

Regarding the MAL, the included studies used different versions for data collection (e.g., MAL-13, MAL-14, MAL-28, MAL-26, MAL-30). Moreover, in some studies only the use of one of the two MAL scales [30] is reported. Correlation coefficients were highest in the cross-sectional study of Narai et al. (ratio MAL AOU 0.84, delta 0.70; ratio MAL QOM 0.79, delta 0.66), who used tri-axial accelerometers and reported correlations between different algorithms. They subtracted the movement counts of the unaffected UL from those of the affected UL (delta count) to estimate the affected UL use after controlling for the effect of the activities of other parts of the body on movement counts [41].

Correlations between accelerometry measures (use ratio) and the ARAT range from 0.15–0.79. The strongest correlation between the ARAT and accelerometry was found by Urbin et al., who used tri-axial accelerometers and calculated use ratio, magnitude ratio and variation ratio (0.79, 0.83, 0.85; *p* < 0.001) [40]. The weakest correlation between median bilateral magnitude values and the ARAT, measured by tri-axial accelerometers, was 0.30 (*p* = 0.04), but a moderate correlation with median magnitude ratio values was found (rs = 0.66, *p* < 0.001) [12]. Correlations between activity counts recorded by uni-axial accelerometers and the ARAT were reported by Lang et al. (0.40) [10].

## Discussion

We aimed to examine whether bilateral accelerometry is suitable to objectively measure arm use in everyday life among stroke patients. We found a variety of correlations between accelerometry data and different measurements. However, the data from the studies included show many differences in methodology. They varied largely with regard to (1) accelerometer devices used, including wearing time and data capture epochs, (2) setting and participants (e.g., severity of motor impairment and time since stroke), and (3) coefficients and cut-off values for an analysis of correlations. These differences make it difficult to summarise the results and comparisons must be considered with caution. Data relating to the type of device, wearing times, and data capture epochs varied reasonably and emphasise the need for standardisation: 12 of the 34 articles included in this review referred to tri-axial accelerometry, wearing times ranged from 24 h to three consecutive days and data capture epochs from one second [12, 18, 31, 37, 40, 44, 45, 48] to one minute [34]. A wearing period of seven consecutive days including a weekend day as recommended by Thiel et al. [54] was not considered in any of the articles included [55]. However, until today, there is no consensus as to how long accelerometer data should be collected in a real-world environment in order to achieve meaningful results on everyday UL use [51, 56, 57]. The question regarding a suitable epoch length has not been systematically studied in adults either [58]. Conclusive evidence for the superiority of uni-axial, bi-axial or tri-axial accelerometers measuring activities of daily living is lacking [6, 23, 58]. While some authors suspect that, under certain circumstances, there is no difference in the validity of different device types [32, 59], others recommend the use of multi-axial accelerometry because they are thought to be more valid [50]. Trost et al., for example, found stronger correlations with multi-axial accelerometers compared to uni-axial accelerometers [51, 58].

Overall, the range of available instruments results in an inconsistent use of outcome measures in studies, thereby limiting the comparability of study results relating to correlations between accelerometry and other measures [22]. Nonetheless, the majority of the studies were able to demonstrate moderate correlations between accelerometer data and other measurements despite of different methodologies.

### Limitations of accelerometry

Beside the described inconsistencies in the data collection methods, accelerometry itself has limitations as well. First, accelerometers do not provide information about the quality of specific activities [18, 31, 60].

Second, wrist-worn accelerometers are unable to distinguish between volitional and non-volitional UL movements (e.g., UL swinging while walking) [41, 50, 61, 62]. As a result, measurements may be influenced by different activities, such as walking, sleeping, driving a car or generally times where the accelerometers are not worn (e.g., Uswatte et al. [24],). Some studies considered these effects as accelerations, others did not. According to some studies walking has a negligible influence on accelerometer ratio variables [24, 40], while Bailey et al., for example, compared the effect of walking phases on the activity level and found that arm swing while walking may constitute cause a substantial part of overall activity in patients with severe paresis [12]. As a standard approach, delta counts (subtracted movement counts of the unaffected UL from those of the affected UL) are recommended to produce more reliable data [41].

Finally, a secondary analysis by Waddell and Lang revealed a high variability between self-reported performance of UL and the use ratio of accelerometers over time [30]. This might indicate that there is a high error potential regarding correlations between other measures and accelerometry.

### Limitations of the review

The systematic approach conducted in this review can be regarded as a key strength: identified articles and relevant results were systematically and independently extracted by two researchers. Furthermore, the literature search was not restricted to validity studies, but also considered articles that used accelerometry as an outcome parameter and reported on correlations between accelerometry and other assessments. For each term, a synonym was used. However, we may have missed relevant articles since the screening of found articles first focused on the articles’ titles and abstracts. Finally, the restriction to full-text availability might represent another limitation.

Additionally to these methodological limitations, one has to consider limitations of the cited literature [14, 23–25, 27, 32], such as a very small sample size or only weakly moderate correlations. Moreover, only few studies report on missing data owing to non-compliance with accelerometer measurements in patients or technical issues, which means there is a lack of information on data quality. Also, several studies did not distinguish between acute and chronic stroke patients. The inconsistencies in accelerometry use limit the comparability of study results. Despite these limitations some studies showed significant correlations between clinical measurements and accelerometry, indicating that accelerometry might be suitable for an objective measurement of daily activity in stroke patients.

## Conclusions

Accelerometry can provide objective information about daily arm use frequency in stroke patients and results correlate moderately with self-reported measures. Collecting these data is helpful for assessing the rehabilitation process, although accelerometry does not supply information about the quality of movements and the specific restrictions on everyday activities of stroke patients. Nonetheless, depending on the context, it is advisable to use both, accelerometer and other clinical measures. When using accelerometry in further research, the use of multi-dimensional accelerometers is preferable because these devices measure multiple levels of motion and there is more evidence regarding the suitability of multi-axial than uni-axial models. However, the variability of the study conditions does not allow to recommend a particular accelerometer device most suitable to measure UL activity.

## Supplementary Information

**Additional file 1.** Search algorithms.

**Additional file 2: Table S1.** Characteristics of included articles. **Table S2.** Methods and accelerations of included articles.

## Data Availability

The datasets used and/or analysed during the current study are available from the corresponding author on reasonable request.

## Abbreviations

ARAT: Action Research Arm Test
FIM: Functional Independence Measure
FMA: Fugl-Meyer Assessment
MAL-AOU: Motor Activity Log Amount of Use
MAL-QOM: Motor Activity Log Quality of Movement
NEADL: Nottingham Extended Activities of Daily Living
NIHSS: National Institutes of Health Stroke Scale
REACH: Rating of Everyday Arm-use in the Community and Home
SIS: Stroke Impact Scale
UE: Upper Extremity
UL: Upper Limb (synonym for upper extremity)
WMFT: Wolf Motor Function Test
