# Supplementary material for: Suitability of accelerometry as an objective measure for upper extremity use in stroke patients
Source: BMC Neurol. 2022 Jun 15;22:220. doi: 10.1186/s12883-022-02743-w (PMC9199226; doi:10.1186/s12883-022-02743-w)
Supplement: Supplementary file 1 — Additional file 1. Search algorithms. [file 12883_2022_2743_MOESM1_ESM.docx]

|  | **PubMed (including Medline)** | **Cochrane Library** | **Scopus** | **PeDro** | **LIVIVO** |
| --- | --- | --- | --- | --- | --- |
| **Search Term** | ((((((((((((Accelerometr*) OR Accelerometry[MeSH Terms]) OR Actigraph*)) AND (((stroke) OR stroke[MeSH Terms]) OR apoplexy))) AND (((Upper extremity) OR Arm activity) OR physical activity))) AND ((paresis) OR Motor impairment))))) | Accelerometr* OR Actigraph* AND stroke OR apoplex*AND “upper extremity” OR “arm activity” OR “physical activity” AND paresis OR “motor impairment” | ((((accelerometr*) OR (actigraph*)) AND ((stroke) OR (apoplex*))) AND (("upper extremit*”) OR ("arm activit*") OR ("physical activit*"))) AND ((paresis) OR ("motor impairment")) | Accelerometr* AND stroke | ((((((((((((Accelerometr*) OR Accelerometry[MeSH Terms]) OR Actigraph*)) AND (((stroke) OR stroke[MeSH Terms]) OR apoplexy))) AND (((Upper extremity) OR Arm activity) OR physical activity))) AND ((paresis) OR Motor impairment))))) |
| **Matches** | n=77 | n=15 | n=389 | n=8 | n=118 |

Additional file 1: Search algorithms
