# Supplementary material for: Suitability of accelerometry as an objective measure for upper extremity use in stroke patients
Source: BMC Neurol. 2022 Jun 15;22:220. doi: 10.1186/s12883-022-02743-w (PMC9199226; doi:10.1186/s12883-022-02743-w)
Supplement: Supplementary file 2 — Additional file 2: Table S1. Characteristics of included articles. Table S2. Methods and accelerations of included articles. [file 12883_2022_2743_MOESM2_ESM.docx]

**Table 1: Characteristics of included articles**

| **Reference** | | **Type of study** | **Aim** | **Setting** | **Sample size** | **Status of stroke** | **Impairment** | **Assessments** |
| --- | --- | --- | --- | --- | --- | --- | --- | --- |
| Bailey et al., 2015^12^ | | Cross-sectional study | To quantify real-world bilateral upper-limp (UL) activity in nondisabled adults and adults with stroke using a recently developed accelerometry-based methodology | Community setting | Non-disabled adults n=74, adults with chronic stroke n=48 | Ischaemic orhaemorrhagic stroke; median time since most recent stroke was 0.9 (interquartile range, IQR: 1.3) years | Lower, moderate and higher impairment (Action Research Arm Test, ARAT=10-48) | ARAT |
| Bailey et al., 2015^46^ | | Cross-sectional study | To characterise affected UL activity and examine potential modifying factors of affected UL activity in community-dwelling adults with chronic stroke | Community setting | 46 | Mild to moderate chronic stroke, median time since stroke 0.9 years (IQR = 1.4) | Mild to moderate unilateral UL weakness, National Institutes of Health Stroke Scale (NIHSS) score 1–3 on item and ability to actively move the affected UL: (ARAT) score of 10–49 | Potential modifying factors, e.g.: motor dysfunction of the affected UL (ARAT), activities of daily living (ADL) status |
| Barth et al., 2020^18^ | | Prospective observational study | To identify and characterise the range of UE motor behaviours during the first week after stroke, using widely accepted clinical measures and also objective quantification using wrist-worn accelerometers | Urban safety net hospital | 25, control group (n=12): hospitalised for non-neurological conditions | Mean days after stroke 4.5 ±1.8 | Wide severity range | UE Fugl-Meyer (UEFM), ARAT, Shoulder Abduction/Finger Extension Test (SAFE), NIHSS |
| Barth et al., 2020^34^ | | Secondary analysis of a single-blind, randomised, parallel dose-response trial | To characterise how accelerometer variables may reflect UL compensatory movement patterns after stroke | In and out of clinic | 78? | Chronic stroke: time since stroke ≥6 months (median 12 months post-stroke) | Mild to moderate severity | ARAT scored by two raters by using video recordings (compensatory movement score) |
| Bhatnagar et al., 2020^47^ | | Cross-sectional study | To determine if clinical evaluations of post-stroke arm function correspond to everyday motor performance indexed by arm accelerometers | Outpatient research centre | 21 (one excluded due to accelerometer recording errors) | Chronic stroke (≥6 months) | Moderate arm impairment based on a Fugl-Meyer (FM) score of 19-50 out of 66 | Fugl-Meyer-Assessment (FMA), ARAT-12, Wolf Motor Function Test (WMFT), Motor Activity Log (MAL) 13, Stroke Impact Scale (SIS) hand motor subscale |
| Chen et al., 2018^36^ | | Validation and psychometric study | To investigate the predictive validity, responsiveness, and minimal clinically important difference of arm accelerometer in real-world activity of patients with chronic stroke | Three medical centres | Pooled data of 82 participants from three previous and ongoing randomised controlled trials for secondary analysis | Chronic stroke: months after stroke were 20.46 ± 13.43 | Mild to moderate impairment, FMA score 24-66) | MAL, SIS, Nottingham Extended Activities of Daily Living (NEADL) |
| Chin et al., 2019^39^ | | Observational, exploratory study | To characterise paretic UL use in people with different levels of impairment 4 weeks post-stroke and to compare accelerometry and direct observational approaches | Subacute rehabilitation hospital | 12 participants; data from one participant with moderate impairment was removed due to extreme difference between accelerometry and observational data (mean difference of 4.2 hours (hrs) during 12 hr of observation). | Early subacute phase post-stroke (≤4 weeks), | Different levels of UL motor capability; mild (FMA score 51-66; n=5), moderate (FMA score 23-50; n=3) and severe (FMA score 0-22; n=4) UL impairment | Direct observation (12 hr of behavioural mapping) in hospital: UL movement, functional UL movement, Non-functional and passive UL movement |
| Connell et al., 2014^40^ | | Observational study | To explore whether a relationship exists between wrist accelerometer activity counts and observed repetitions | Rehabilitation centre research laboratory,  community setting | 13 community-dwelling participants | On average 8.5 years after stroke | ARAT-score (4-57; median=56), only three participants had severe UL impairment | Rating of Everyday Arm-use in the Community and Home Scale (REACH) that captures affected UL use outside of the clinical setting.  Total session time and time spent completing each GRASP exercise |
| Gebruers et al., 2008^27^ | | Prospective study | (1) To investigate the validity of actigraphy as an objective measurement of motor deficits in acute stroke patients, and  (2) to determine the sensitivity, specificity and diagnostic accuracy of actigraphy to score motor activity compared to NIHSS and FMA | Hospital | 39 | Acute ischaemic stroke, <7 days after onset | Not reported, NIHSS median score =7, NIHSS interquartile range =11, FMA median score =44, FMA interquartile range =55 | NIHSS and FMA arm section |
| Gebruers et al., 2011^41^ | | Longitudinal observational study | To investigate the disuse hypothesis as an explanation for UL oedema in patients with stroke | Hospital | 139 | Acute hemiparetic stroke, (<7 days after stroke onset), ischaemic or haemorrhagic stroke | Not reported | Baseline (t0), after 1 week (t1), at 1 month (t2), at 3 months (t3); NIHSS (t0-t3), FMA (t0-t3), modified Rankin Scale (mRS) (t3) |
| Gebruers et al., 2013^48^ | | Prospective study | To assess the predictive value of accelerometer-based measurements of upper limb activity in patients with acute stroke | Not reported | 129 acute stroke patients and 24 control patients | Acute stroke; <7 days after onset (median: 1 day, range: 1–6 days) | Acute stroke; <7 days after onset (median: 1 day, range: 1–6 days) | FMA UL section at study entry, NIHSS, mRS at 3 months follow-up |
| Gebruers et al., 2014^49^ | | Prospective study | To investigate the clinical predictive value of the FMA arm score and the UL activity assessed by accelerometers in patients with hemiparesis after acute stroke | See Gebruers et al., 2013^48^ | See Gebruers et al. 2013^48^ | See Gebruers et al. 2013^48^ | See Gebruers et al., 2013^48^ | FMA, mRS at 3 months follow up |
| Lang et al., 2007^10^ | | Randomised controlled trial | (1) To determine the amount of UL use in people with hemiparesis post-stroke during their inpatient rehabilitation stay, and  (2) to examine the relationships between UL use and impairments and activity limitations | Inpatient rehabilitation facility | 34 subjects and 10 healthy control subjects | Mean time since stroke =9.3 days) (9.3 +/- 4.2) | Mild-to-moderate acute hemiparesis | ARAT, WMFT, Functional Independence Measure (FIM) |
| Narai et al., 2016^43^ | | Cross-sectional study | (1) To examine whether objectively measured real-world UL movement is associated with the amount of use of the affected UL in older participants with acute or subacute stroke, and  (2) to examine whether the real-world UL movement is associated with the extent of impairment of upper and lower extremities | Inpatient rehabilitation facility | 19 older participants (>65 years) | Acute or subacute stroke 17 (7) days after stroke; cerebral ischaemic: (n=13); haemorrhagic (n=6) | Not reported | MAL, NIHSS, Brunnstrom Recovery Stage (BRS), Simple Test for evaluating Hand function (STEF) and FIM |
| Reiterer et al. 2008^35^ | | Clinical trial | To evaluate actigraphy as a tool to objectify the recovery process after motor paresis due to stroke | Hospital | 38 participants (only 28 patients were considered in the statistical analysis) | Acute stroke: 24-36 hrs; 5-7 days, 3 months and 6 months after stroke | Mild deficits | Motoricity Index (MI), NIHSS, Scandinavian Stroke Scale, (SSS) Rankin Scale (RS), Barthel Index (BI) |
| Simpson et al., 2013^44^ | | Cross-sectional study | (1) To develop a classification scale called the REACH scale that captures affected UL use outside the clinical setting, and  2) to assess the inter-rater reliability and validity of the new scale | Individuals from community stroke recovery groups and former inpatients from a local rehabilitation hospital | 96 community-dwelling participants; a total of 68 were included in the final accelerometer analysis | 7.8 years post-stroke (SD 3.3) | Mild to moderate stroke severity (NIHSS) and full range of UL impairment levels from mild to severe (Chedoke-McMaster arm and hand subscale range 2–14). | REACH scale, MAL-14, SIS subscale (hand), ARAT, Chedoke-McMaster Stroke Assessment |
| Thrane et al., 2011^45^ | | Cross-sectional study | To assess the effect of arm motor impairment on actual arm use in the early post-stroke period and explore its association with self-care dependency | Hospital | 31 patients recruited from two trials from stroke units | 1-30 days after stroke (mean time since stroke onset 10.6 days (SD 6.0) | NIHSS score 0-20 (median 3) | FMA |
| Urbin et al., 2015^50^ | | Exploratory study | To identify the acceleration characteristics that have a stable association with UL function and sensitivity to within-participant fluctuations in function over multiple sessions of task-specific training | Clinic | 27 | Chronic hemiparesis >6 months after stroke | Mild to moderate  Inclusion criteria:  Movement of the paretic UL as defined by a score of ≥10 on the ARAT | Hand Function Subscale, SIS, MI, ARAT |
| Urbin et al., 2015^42^ | | Before-After Observational Study | (1) To determine if acceleration metrics derived from monitoring outside of treatment are responsive to change in UE function  (2.1) To compare metric values during task-specific training and while in the free-living environment  (2.2) To establish metric associations with an in-clinic measure of movement capabilities | Inpatient hospital (primary purpose); outpatient hospital (secondary purpose) | 8 participants from inpatient hospital (primary purpose); 27 participants from outpatient hospital (secondary purpose) | Primary purpose: participants with <30 days post-stroke  Secondary purpose: participant with ≥6 months post-stroke | Mild to moderate | ARAT |
| Uswatte & Taub, 2005^19^ | | Validation study | To evaluate the reliability and validity of accelerometry for measuring UL rehabilitation outcome | At home, seven out-of-town patients stayed in hotels | 10 stroke patients of Consecutive Constraint-Induced Movement Therapy (CIMT) and 10 volunteer community residents with stroke; one in each group was excluded | Chronic stroke,  >1 year post-stroke | Mild to moderate motor impairment | MAL |
| Uswatte et al., 2005^14^ | | Validation study | To examine the reliability and validity of the original, 14-item version of the MAL in 2 independent samples of individuals who were >1 year after stroke | Outside the laboratory | 27 | Chronic stroke (>1 year after stroke | Mild to moderate impairment of UL hemiparesis | MAL-14, MAL before (test 1) and after (test 2) AutoCITE therapy |
| Uswatte et al., 2006^25^ | | Validation study (EXCITE trial) | To examine the psychometric properties of an objective method for assessing real-world arm activity in a large sample with subacute stroke | Community | 169 | Subacute stroke patients 3 to 9 months post stroke | Mild to moderate impairment of hemiparetic arm | Actual Amount of Use Test (AAUT), MAL, SIS Mobility scale |
| Uswatte et al., 2006^31^ | | Clinical trial (EXCITE trial) | To study the MAL’s reliability and validity for assessing real-world Quality Of Movement (QOM) scale and Amount of Use (AOU) scale of the hemiparetic arm in stroke survivors | Outside the laboratory | 222:  106 (treatment),  116 (control) | Subacute stroke 3 to 12 months post stroke | Mild to moderate paresis | MAL-30, SIS Mobility scale |
| Uswatte & Qadri 2009^37^ | | Validation study | To evaluate psychometric properties of the Functional Arm Activity Behavioral Observation System for measuring hemiparetic arm use | Hospital or at home | Study 1: n=9  Study 2: n=8 (accelerometer data of two participants were not available) | Brain injury >1 year prior to study entry | Mild to moderate UL hemiparesis | Videotapes (Functional Arm Activity Behavioral Observation System (FAABOS)) in 2-second blocks to count more-impaired arm activity |
| Van der Pas et al., 2011^6^ | | Cross-sectional concurrent validity study | To study the validity of accelerometry in the assessment of arm activity of patients with impaired arm function after stroke | Rehabilitation centre | 45 | On average 8.5 years after stroke | Different stages after stroke, impairment not reported | MAL-26, SIS subscale Hand Function, SIS Mobility scale after wearing accelerometers |
| Waddell & Lang 2018^33^ | | Brief report, secondary analysis of a phase II randomised, parallel, dose-response UL movement trial | To compare self-reported with sensor-measured UL performance in daily life for individuals with chronic (≥6 months), UL paresis post-stroke | Outpatient rehabilitation | 64 participants (21 excluded participants had either accelerometer recording errors, missing self-report data or withdrew from the study) | Chronic stroke (≥6 months) | Not reported | MAL-28 AOU |
| Wang et al., 2011^38^ | | Validation and clinimetric study | To investigate the criterion-related validity, responsiveness and clinically important differences of the ABILHAND questionnaire in patients with stroke | Medical centres | 51 | Months after stroke 17.57 ± 13.43 | Not reported | ABILHAND |
| **Systematic Reviews** | | | | | | | | |
| Connell et al., 2012^28^ | Systematic review | | To review the psychometric properties and clinical utility of UL ability in neurologic conditions to provide recommendations for practice | Not reported | The searches identified 31 measures of the UL.  However, 19 measures were subsequently rejected. Four were composite measures where it was not possible to extract information on subscales of the UL, 2 measured other constructs such as strength, 12 did not meet the clinical utility criteria, and 1 had only 1 psychometric property tested; 7 measures evaluated ADL  Relevant articles: Uswatte et al., 2005^24^, Uswatte et al., 2005^14^ | Stroke, but also Parkinson’s disease, multiple sclerosis and other neurologic conditions | Not reported | MAL, ABILHAND, WOLF, ARAT |
| Gebruers et al., 2010^23^ | Systematic review | | To assess the clinimetric properties and clinical applicability of different accelerometry-based measurement techniques in persons with stroke | Different settings (laboratory, clinic, home) | 25 articles (4 randomized controlled trials) were included. The search yielded 9 articles concerning the measurement of the UL activity  Relevant articles: Uswatte et al., 2005^14^, Uswatte et al., 2006^31^, Uswatte et al., 2006^25,^ Uswatte et al., 2005^24,^ Gebruers et al., 2008^27^ | Acute, subacute and chronic stroke | Impairment/ measurement of UL and lower extremity (activities):  severely and moderately impaired patients | MAL, SIS, NIHSS, FMA Arm section score, MI, SSS, Rankin Scale, BI, WMFT, FIM, ARAT |
| Hayward et al., 2015^52^ | Review | | To explore the feasibility of accelerometers to measure UL use after stroke and discuss the translation of this measurement | Different settings (laboratory, clinic, home) | Information about the screening process of the literature search is missing.  Relevant articles: Lang et al., 2007^10^, Reiterer et al., 2008^35^, Gebruers et al., 2014^49^, Thrane et al., 2011^45^, Uswatte et al., 2005^24^, Uswatte et al., 2006^31^, Van der Pas et al., 2011^6^, Simpson et al., 2013^44^ | Any stage of recovery | Not reported | Accelerometers: Actigraph (Actigraph, Pensacola FL), and Actical (Mini Mitter Co, Bend OR) units  Human-observed purposeful repetitions, FIM, FMA of UL function, ARAT, REACH Scale |
| Lang et al., 2013^5^ | Review | | To provide a comprehensive approach for assessing the UE after stroke | In- and outside the laboratory/ clinic | Relevant articles:  Reiterer et al., 2008^35^, Uswatte et al., 2006^25^, Lang et al., 2007^10^, Thrane et al., 2011^45^, Gebruers et al., 2010^23^ | Not reported | Different impairment levels | Correlations reported between accelerometry and MAL-QOM, MI, FMA, ARAT, WMFT, FIM, BI, SIS |
| Lang et al., 2017^51^ | Protocol about accelerometry methods (Preparing, Placement, Download of data) | | To detail a methodology for measuring upper limb performance in daily life using accelerometers worn on the wrists | In- and outside the laboratory/ clinic | Relevant articles:  Lang et al., 2013^5^, Gebruers et al., 2010^23^; Uswatte et al., 2005^24^, Uswatte et al., 2006^25^, Lang et al., 2007^10^ | Not reported | Not reported | Accelerometer measures: hrs of use, use ratio, magnitude ratio, bilateral magnitude |
| Lemmens et al., 2012^22^ | Systematic review | | To identify, evaluate and categorise instruments reported to be valid and reliable, assessing arm-hand performance at the ICF activity level in patients with stroke or cerebral palsy | In- and outside the laboratory/ clinic | Capacity (18 instruments, 13 for stroke patients), perceived performance (9 instruments, 4 for stroke patients) and actual performance (3 instruments, 2 for stroke patients). Content of the instruments differed widely regarding the ICF levels measured, assessment of the AOU versus the quality of use, the inclusion of unimanual and/or bimanual tasks and the inclusion of basic and/or extended tasks  Relevant articles: Gebruers et al., 2010^23^, Uswatte & Quadri 2009^37^ | Not reported | Not reported | Relevant for present review: ARAT, WMFT, ABILHAND, MAL, FAABOS, accelerometry |
| Noorkõiv et al., 2014^53^ | Systematic review | | To identify and summarise publications that have reported clinical applications of UL accelerometry for stroke within free-living environments and to make recommendations for future studies | Free-living hospital or home setting | 8 articles included, correlation between clinical assessments and accelerometry was tested in five studies  Relevant articles: Uswatte et al., 2006^31^, Lang et al., 2007^10^, Thrane et al., 2011^45^, Van der Pas et al., 2011^6^, Wang et al., 2011^38^ | Heterogeneous range of UL impairment | There was a tendency to moderate to mild impairment. Time since stroke varied across a wide range | FMA subscale for UE, ARAT, WMFT, FIM for motor and UE function,  MAL, SIS Hand Function Subscale, ABILHAND, NEADL |
| Note: **AAUT** – Actual Amount of Use Test, **ABILHAND** – Action Observation Training and Motor Imagery Therapy, **ADL** – Activities of Daily Living, **AMR** – Arm Movement Ratio, **ARAT** – Action Research Arm Test, **AOU** – Amount of Use, **BI** – Barthel Index, **BRS** – Brunnstrom Recovery Stage, **FAABOS** – Functional Arm Activity Behavioral Observation System, **FIM** – Functional Independence Measure, **FMA** – Fugl-Meyer Assessment, **hr(s)** – hour(s), **MAL** – Motor Activity Log, **MI** – Motricity Index, **mRS** – modified Rankin Scale, **NEADL** – Nottingham Extended Activities of Daily Living, **NIHSS** – National Institutes of Health Stroke Scale, **PIM** – Proportional Integrating Measure, **QOM** – Quality Of Movement, **REACH QOM** – Rating of Everyday Arm-use in the Community and Home, **RS** – Rankin Scale, **SAFE** – Shoulder Abduction/Finger Extension Test, **SIS** – Stroke Impact Scale, **SSS** – Scandinavian Stroke Scale, **STEF** – Simple Test for Evaluating Hand Function, **TAT** – Time Above Threshold; **UL** – Upper limb (synonym for upper extremity), **WMFT** – Wolf Motor Function Test | | | | | | | | |

**Table 2: Methods and accelerations of included articles**

| **Reference** | **Setting** | **Accelerometer** | **Wearing time** | **Accelerometry measures** | **Epoch** | **Definition of Correlation** | **Results** |
| --- | --- | --- | --- | --- | --- | --- | --- |
| Bailey et al., 2015^12^ | Community setting | Tri-axial accelerometer (GT3X+ Activity Monitors Actigraph, Pensacola, FL) | 25-26 hrs | 30 Hz; band-pass filtered data between frequencies of 0.25-2.5 Hz; down-sampled data to 1 Hz  (1) The intensity of bilateral UL activity (i.e., bilateral magnitude)  (2) The contribution of both ULs to activity (magnitude ratio) | 1 second | Weak: =0.30,  moderate: =0.66,  significance alpha <0.05 | Motor capability (ARAT score) was weakly correlated with median bilateral magnitude values (=0.30, p=0.04) and moderately correlated with median magnitude ratio values ([ρ](https://de.wikipedia.org/wiki/Rho)=0.66, p<0.001) |
| Bailey et al., 2015^46^ | Community setting | Tri-axial accelerometer (GT3X Activity Monitors Actigraph; Pensacola, FL) | 25 hrs | 30 Hz; duration of affected and unaffected UL, ratio of affected-to-unaffected UL activity | 1 second | Weak: [ρ](https://de.wikipedia.org/wiki/Rho)<0.30, moderate: [ρ](https://de.wikipedia.org/wiki/Rho)=0.30-0.60,  strong: [ρ](https://de.wikipedia.org/wiki/Rho)≥0.60,  significance alpha <0.05 | Correlations between motor dysfunction of affected UL measured by ARAT and activity ratio measured by accelerometry [ρ](https://de.wikipedia.org/wiki/Rho)=0.63*, and hrs of affected UL activity [ρ](https://de.wikipedia.org/wiki/Rho)=0.49* |
| Barth et al., 2020^18^ | Hospital | Tri-axial accelerometer (ActiLife Actigraph, Pensacola, FL | 24 hrs | 1 Hz, total duration of movement of each limb, duration of unilateral and bilateral movements, use ratio (activity ratio of hours of movement of the paretic limb to the non-paretic limb) | 1 second | Significance level at 0.01 | Moderate correlations between use ratio and  ARAT: *r*=0.65**  UEFM: *r*=0.69**  SAFE: *r*= -0.69**  NIHSS Motor Arm: *r*= -0.69** |
| Barth et al., 2020^34^ | In and out of clinic | Tri-axial accelerometer (wGT3X+, Actigraph, Pensacola, FL) | 24 hrs (1.5 hrs in-clinic and 22.5 hrs out-of-clinic) | 30 Hz, time, magnitude and variability of UL movement, jerk asymmetry index and the spectral arc length (quality/smoothness of movement) | 1 second | Spearman’s coefficients:  low: [ρ](https://de.wikipedia.org/wiki/Rho)≥0.25,  moderate: [ρ](https://de.wikipedia.org/wiki/Rho)=0.26 to 0.50,  good: [ρ](https://de.wikipedia.org/wiki/Rho)=0.51 to 0.75,  excellent: [ρ](https://de.wikipedia.org/wiki/Rho)> 0.75, statistical significance at α< 0.05 | ARAT  Out-of-clinic measures and  variability of bilateral movement [ρ](https://de.wikipedia.org/wiki/Rho)= -0.35, p<0.01  Isolated use of the nonparetic limb to compensatory movement score [ρ](https://de.wikipedia.org/wiki/Rho)=0.61, p<0.0001  Use ratio to the compensatory movement score [ρ](https://de.wikipedia.org/wiki/Rho)=−0.57, p=0.18 Jerk Asymmetry Index to compensatory movement scores [ρ](https://de.wikipedia.org/wiki/Rho)= -0.19, p=0.09 Spectral arc length of the paretic limb to compensatory movement  scores [ρ](https://de.wikipedia.org/wiki/Rho)=0.29, p<0.01 |
| Bhatnagar et al., 2020^47^ | Outpatient research centre | Tri-axial accelerometer (GT3X+ Actigraph. Pensacola, FL) | 72 hrs (including showering and sleeping) | Quantifications of movement intensity (magnitude) and duration of arm use | 1 second | Significance level of 0.05 | Mean magnitude ratio and  FM ρ=0.60, p<0.01,  WMFT functional score ρ=0.59, p<0.01  ARAT ρ = 0.50, p<0.05  Hrs of use and  MAL AOU ρ=0.58, p<0.01  MAL QOM ρ=0.61, p<0.01  Total paretic hrs did not correlate with the FMA, WMFT or ARAT, and intensity variables did not correlate with the MAL or SIS |
| Chen et al., 2018^36^ | Three medical centres | MicroMini-Motionlogger activity monitor (Ambulatory Monitoring, NY) | 72 hrs (except bathing) before and immediately after intervention | 10 Hz summed over a 1-minute epoch,  affected arm activity | 60 seconds | Pearson’s *r* value; low: 0-0.25, fair to moderate: 0.25-0.5 moderate to good: 0.5-0.75, good to excellent: >0.75 | Fair to moderate correlations between the arm accelerometer and the three measurements; MAL [*r*](https://de.wikipedia.org/wiki/Rho)=0.47, SIS *r*=0.42 and NEADL *r*=0.34; moderate to good correlations between MAL QOM and accelerometer *r*=0.57 |
| Chin et al., 2019^39^ | Subacute rehabilitation hospital | Tri-axial accelerometer (GT3X+, Activity Monitors ActiGraph LLC, Pensacola, FL) | 24 hrs (except showering) | 60 Hz, vector magnitude of ≥2 activity counts for at least 1 s, ratio of duration of paretic/nonparetic UL use (use ratio); ratio of magnitude of paretic/nonparetic UL use (magnitude ratio); sum of vector magnitude of bilateral UL use (bilateral magnitude); and ratio of variability (standard deviation) of paretic/nonparetic UL use (variation ratio) | 1 second | Moderately strong correlations ICC=0.88  significance was set at p<0.05 | Accelerometry and direct observation were significantly positively correlated (p < .001) across the 12 hrs of direct observation (ICC = 0.882, 95% CI [0.64, 0.97]) |
| Connell et al., 2014^40^ | Rehabilitation centre research laboratory,  community setting | Tri-axial Actical® accelerometer | During the exercise session, mean active time in session =35.9 min (SD 6.9) | 32 Hz, the activity counts were summed for both affected and unaffected UL, epochs with no activity counts were filtered out | 15 seconds | Moderate correlation: [ρ](https://de.wikipedia.org/wiki/Rho)=0.63,  significance value p <0.05 | No correlation between REACH scale and accelerometer counts reported; purposeful repetitions and activity counts significantly correlated ([ρ](https://de.wikipedia.org/wiki/Rho)=0.63, p<0.05); no significant correlation between total repetitions and accelerometer activity counts ([ρ](https://de.wikipedia.org/wiki/Rho)=0.35, p=0.24)  Time spent active varied between raters’ observations (63.8%) and accelerometer measures (75.7%) |
| Gebruers et al., 2008^27^ | Hospital | Uni-axial octagonal basic motion loggers (Ambulatory Monitoring Inc; Ardsley, NY, USA) | 48 hrs, because the recordings did not start at the same time of day, the 48-hr data were averaged into a 24-hr data set. Inactive periods were checked against the event log and deleted when motion logger was actually removed | 2- to 3-Hz low-pass filter; Zero Crossing Mode (ZCM), Time Above Threshold (TAT), and Proportional Integrating Measure (PIM), Activity of Impaired Arm (AIA) ratio | 1 second, summed 1,800 1-second epoch scores for each half hour | Moderate: [ρ](https://de.wikipedia.org/wiki/Rho)=0.59, strong: [ρ](https://de.wikipedia.org/wiki/Rho)=0.81  significance p <0.05 | Moderate but highly significant correlations (Spearman’s rho) between actigraphic recordings and total NIHSS (ratio [ρ](https://de.wikipedia.org/wiki/Rho)= –0.59 and AIA [ρ](https://de.wikipedia.org/wiki/Rho)= –0.75; p=0.001 and FMA ratio [ρ](https://de.wikipedia.org/wiki/Rho)= 0.54 and AIA [ρ](https://de.wikipedia.org/wiki/Rho)=0.69; p=0.001 scores; strong and negative correlation between NIHSS and FMA [ρ](https://de.wikipedia.org/wiki/Rho)=–0.81; p= 0.001;  Correlations between actigraphy and the stroke scales were best when mean activity variables were used |
| Gebruers et al., 2011^41^ | Hospital | Uni-axial octagonal motion logger (Ambulatory Monitoring Inc., NY, USA) | Two 48‑hr periods with a 1-week interval | 2–3 Hz low-pass filter; total activity, measured as a total sum of raw counts, and calculated ratio variable (t0; t1) PIM | Not reported | Not reported | Ratio t0  and NIHSS t0-t3 [ρ](https://de.wikipedia.org/wiki/Rho)=-0.25**-  -0.37**  and FMA t0-t3 [ρ](https://de.wikipedia.org/wiki/Rho)=0.28**-0.50**  and mRS t3 [ρ](https://de.wikipedia.org/wiki/Rho)=-0.13  Ratio t1  and NIHSS t0-t3 [ρ](https://de.wikipedia.org/wiki/Rho)=-0.43- -0.49**  and FMA t0-t3 [ρ](https://de.wikipedia.org/wiki/Rho)=0.61-0.71  and mRS t3 [ρ](https://de.wikipedia.org/wiki/Rho)=-0.49**  Activity paretic arm t0  and NIHSS t0-t3 [ρ](https://de.wikipedia.org/wiki/Rho)=-0.52**-  -0.61**  and FMA t0-t3 [ρ](https://de.wikipedia.org/wiki/Rho)=0.50**-0.73**  and mRS t3  [ρ](https://de.wikipedia.org/wiki/Rho)=-0.61**  Activity paretic arm t1 and NIHSS t0-t3 [ρ](https://de.wikipedia.org/wiki/Rho)=-0.55**-  -0.61**  and FMA t0-t3 [ρ](https://de.wikipedia.org/wiki/Rho)=0.59-0.71**  and mRS t3  [ρ](https://de.wikipedia.org/wiki/Rho)= -0.59** |
| Gebruers et al., 2013^48^ | Not reported | Uni-axial octagonal basic motion loggers (Ambulatory Monitoring Inc; Ardsley, NY, USA) | 48 hrs averaged to a 24-hr dataset, inactive periods were matched to the event log and deleted when motion logger was removed (e.g., when showering or MRI scanning) | Amount of Activity (e.g., AIA), ratios (e.g., AMR), activity of non-impaired arm, ANIA), PIM | 1 second, 1,800 one- second epoch scores for each half hr | Not reported  Significance p<0.01 | Correlation between AIA and  mRS ρ= -0.59  FMA ρ=0.70  NIHSS ρ= -0.68  Ratio and  mRS ρ= -0.48  FMA ρ=0.60  NIHSS ρ=- 0.51  The sensitivity and specificity for the ratio and AIA are 0.85 and 0.75, respectively (area under curve [AUC] = 0.84) and 0.80 and 0.77 (AUC = 0.87) |
| Gebruers et al., 2014^49^ | See Gebruers et al., 2013^48^ | See Gebruers et al. 2013^48^ | See Gebruers et al., 2013^48^ | See Gebruers et al., 2013^48^ | See Gebruers et al., 2013^48^ | Not reported | Baseline and outcome variables at 3‑month follow-up:  AIA and:  FMA3 ρ =0.70,  mRS3 ρ =-0.60  Ratio and  FMA3 ρ =0.59  mRS3 ρ =-0.48 |
| Lang et al., 2007^10^ | Inpatient rehabilitation facility | Uni-axial, bilateral accelerometers (model 7164-2.4 Activity Monitors, MTI Health Services, Fort Walton Beach, FL) | 24 hrs except for times when devices would be exposed to water (e.g., personal hygiene) | Activity counts (summation of all accelerations recorded during an epoch) | 2 seconds | Low: ≤0.25, fair: 0.26-0.50, good: 0.51-0.75, excellent:>0.75  >0.33, significance: p<0.05,  >0.42, significance: p<0.01 | Correlations between activity measures and: ARAT ρ= 0.40,  WMFT Function ρ=0.62, WMFT Time ρ =-0.65,  FIM Motor ρ=0.67  FIM UE ρ= 0.58 |
| Narai et al., 2016^43^ | Inpatient rehabilitation facility | Three accelerometers on each wrist (tri-axial, bilateral), 1 on the waist (uni-axial) | 24 hrs (except bathing and showering) | 20 Hz, magnitude accelerations, delta counts (subtracted movement counts of the unaffected UL from those of the affected UL) | 600 seconds | p value < 0.05 | Unilateral accelerometer counts of affected arm and  MAL AOU r=0.58  MAL QOM r=0.55  (p< 0.05)  NIHSS r=-0.69  BRS r=0.64  STEF affected side r=0.67, unaffected side r=0.75  FIM Motor r=0.70 (p<0.01)  Ratio  MAL AOU r=0.84  MAL QOM r=0.79  NIHSS r=-0.60  BRS r=0.77  STEF affected side r=0.86, (p<0.01) unaffected side r= 0.54  FIM Motor r= 0.50 (p<0.05)  Delta  MAL AOU r=0.70  MAL QOM r=0.66 (p<0.01)  NIHSS r=-0.27  BRS r=0.52  STEF affected side r=0.74  (p<0.01), unaffected side r=0.28  FIM Motor r=0.17 |
| Reiterer et al., 2008^35^ | Hospital | Tri-axial actigraphs (Actiwatch™, Cambridge Neurotechnology, UK) | 24 hrs at four different times during the course of rehabilitation 24-36 hrs, 5‑7 days, 3 months and 6 months | Total Activity Scores (TAS) of impaired (TASi) and non-impaired arm separately (TASni) | 15 seconds | Not reported | Correlation between TASi and  MI 24-36 hrs r=0.44 (p=0.01),  MI 5-7 days r=0.5 (p=0.01),  SSS 24-36 hrs r=0.43 (p=0.046),  SSS 5-7 days r=0.53 (p=0.01)  NIHSS no significant correlations  BI 26-36 hrs ρ =0.48 (p=0.03)  BI 5-7 days ρ =0.64 (p<0.01)  RS 24-36 hrs ρ = -0.57 (p<0.07)  RS 5-7 days  ρ = -0.60 (p<0.01)  None of the assessment correlated significantly with TAS at 3 months and 6 months after stroke |
| Simpson et al., 2013^44^ | Individuals from community stroke recovery groups and former inpatients from a local rehabilitation hospital | ActicalTM accelerometers | 72 hrs, during waking hrs | Average daily activity counts of the affected UL | Not reported | Not reported | The REACH scale scores were more strongly correlated to the MAL (ρ=0.94) than the affected UL activity counts (ρ=0.61). |
| Thrane et al., 2011^45^ | Hospital | Uni-axial ActiGraph GT1M Accelerometers (ActiGraph Inc.,  Pensacola, USA) | 24 hrs, data when removing accelerometers (travelling by motor vehicle and sleeping) was excluded | AMR, ratio of arm use duration between the more and less affected arm | 2 seconds | Poor: <0.20, fair: 0.21-0.40, moderate: 0.41–0.60, good: 0.61-0.80 very good: >0.81  p-value: <0.05 | FMA of the more affected arm was strongly associated with AMR r=–0.85 (p>0.001), with the duration of the more affected arm movement measured with the accelerometer ρ=0.60 (p<0.001) |
| Urbin et al., 2015^50^ | Clinic | Tri-axial ActiGraph 6 | During seven sessions of task-specific training that take place 4 times per week for a total of 8 weeks  Raw accelerometer data were truncated to 90 minutes for each session | 30 Hz  Activity counts, Use ratio, Magnitude ratios and Variation ratios | One second | Weak: r≤0.29,  moderate: ρ=0.30-0.59,  strong: ρ≥0.60  ρ>0.38 significance:  p<0.05  ρ>0.48 significance p<0.01 | Median paretic and bilateral acceleration did not correlate with ARAT score at any session  Ratio use and ARAT  session 1 ρ=.43*  session 2 ρ=.15  session 3 ρ=.39*  session 4 ρ=.43*  session 5 ρ=.56*  session 6 ρ=.57*  session 7 ρ=.51*  Bilateral variability  session 1 ρ=.54*  session 2 ρ=61*  session 3 ρ=.34  session 4 ρ=.56*  session 5 ρ=.37  session 6 ρ=.42*  session 7 ρ=.46* |
| Urbin et al., 2015^42^ | Inpatient hospital (primary purpose); outpatient hospital (secondary purpose) | Tri-axial, solid-state digital accelerometer | **Inpatient sample:** 22 hrs outside of treatment before and after multiple sessions of task-specific training  **Outpatient sample:**  During a single session of task-specific training and the subsequent 22 hrs outside of clinical settings | 30 Hz  Activity counts, Use ratio, Magnitude ratio, Variation ratio, median paretic UL acceleration magnitude, paretic UL acceleration variability | 1 second | Strong: ρ≥0.60  significance alpha <0.05  ρ>0.48 significance =p<0.001 | ARAT score measured in clinic and the metrics quantified from the free-living environment were strong:  Use ratio (ρ=0.79, p<0.001)  Magnitude ratio (ρ=0.83, p<0.001)  Variation ratio (ρ=0.85, p<0.001)  Median paretic UL acceleration magnitude (ρ=0.75, p<0.001)  Paretic UL acceleration variability (ρ=0.73, p<0.001) |
| Uswatte et al., 2005^24^ | At home; seven out-of-town patients stayed in hotels | Two-axial accelerometer, (Model 71256 Activity Monitors) worn on each arm, chest and more affected leg | 72 hrs, during all waking hours (except washing, sleeping, riding in a car, not wearing the accelerometers) Appropriate periods were discarded | 10 Hz  Ratio summary variable (ratio of more- to less-impaired arm threshold-filtered recordings): duration of movement as a percentage of the recording period | 2 seconds | Strong: r>0.5 | Ratio summary variable and MAL (period 1) *r*=0.74 (p<0.001), recalculated with observations in the IQR of period 1 ratio summary variable values were still meaningful (*r=*.51, p=0.14; n=10), with respect to change from period 1 to 2, the correlation between the ratio summary variable and MAL was *r* =0.71 (p=0.001). |
| Uswatte et al., 2005^14^ | Outside the laboratory | Two-axial accelerometers (Manufacturing Technologies Inc., Fort Walton Beach, FL) | 72 hrs | Ratio of more- to less-impaired arm accelerometer recordings | Not reported | Not reported | Correlations between pre-to-posttreatment change scores on the accelerometer recordings and participant QOM *r* =0.70  caregiver MAL QOM  *r* =0.73  caregiver MAL AOU  *r* =0.91 (p*=*0.01)  QOM scores were strongly correlated with corresponding changes in both other measures of quality of more-impaired arm movement (e.g., caregiver QOM; *r=*0.70) and amount of more-impaired arm activity (e.g., accelerometry, *r=*0.91, caregiver AOU, *r=*0.73, patient AOU *r=*0.80). |
| Uswatte et al., 2006^25^ | Community | Two-axial accelerometers (Manufacturing Technologies Inc., Fort Walton Beach, FL) | Two 72‑hr periods (period 1 before treatment/no treatment interval and period 2 after treatment/no treatment interval), during all waking hrs, except when washing; segments from accelerometer recordings when subjects were thought to have accelerometry was removed | 10 Hz  Ratio of impaired-arm to unimpaired-arm, impaired-arm summary variable, unimpaired-arm summary variable | 2 seconds | Weak: 0.1,  moderate: 0.3, strong: 0.5 | Baseline: ratio of more-impaired to less-impaired arm accelerometer recordings and AAUT (*r*=0.60) and MAL scores (*r*=0.52, p*=*0.001) respectively, SIS Mobility scale *r* =0.16 (p=0.05) |
| Uswatte et al., 2006^31^ | Outside the laboratory | Two-axial accelerometers (Manufacturing Technologies Inc., Fort Walton Beach, FL) | 72 hrs during all waking hours, except when they were in contact with water | 10 Hz  Ratio of impaired-to-unimpaired arm movement | 2 seconds | Weak: 0.1,  moderate: 0.3, strong: 0.5 | Multitrait-multimethod validation  Correlation between accelerometry ratio and  MAL-QOM *r=*0.52 p=0.01  MAL-AOU *r=*0.47 p=0.01  Less-impaired arm accelerometer recordings and  MAL-QOM *r=*0.14  MAL-AOU *r=*0.14  Correlation was stronger in patients with paresis of their dominant arm (QOM:  *r =*0.59; AOU: *r=* 0.56) than in others (QOM: *r=*0.34; AOU: *r=*0.28). |
| Uswatte & Qadri 2009^37^ | Hospital or at home | Two-axial accelerometer (Model 71256 Activity Monitors; Manufacturing Technologies Inc., Fort Walton Beach, FL) worn on each arm, the chest and a leg | Study 1: 15 minutes in hospital or at home  Study 2: 72 hrs at home | Raw acceleration values; ratio of more-impaired arm to less-impaired arm threshold-filtered accelerometer recordings | 2 seconds | Correlation coefficients > 0.5 are considered strong | The convergent validity of FAABOS was supported by a strong correlation between the FAABOS ratings and the ratio of more- to less-impaired arm accelerometer recordings for data from both studies combined, *r* (14) = 0.55, p<.05. |
| Van der Pas et al., 2011^6^ | Rehabilitation centre | Tri-axial accelerometer (water resistant, Actiwatch AW7a) | 72 hrs, patients documented moments of going to sleep and getting up after sleeping (night-time data was discarded);  For 19 persons, the assessment included weekdays only, whereas 26 persons had at least 1 weekend day included. | 32 Hz, Activity counts: Unilateral (activity of the affected arm) and bilateral accelerometry (ratio between the activity of the affected and nonaffected arm); calculated AMR  1) The total sum of accelerometer counts during waking hrs divided by the number of waking hours.  (2) Bilateral arm activity: the ratio of the sum of daytime acceleration of the impaired arm to unimpaired arm. | 1 second period, counts per time unit (2 seconds) | Not reported | Bilateral arm activity (mean of 2 arms): MAL-26 AOU Scale ρ =0.37, p <0.01, MAL QOM Scale: ρ =0.43, p<0.005; SIS Hand Function Subscale: ρ=0.43, p<0.005;  AMR: MAL-26 AOU Scale ρ =0.60, p<0.001; MAL-26 QOM Scale ρ =0.66, p<0.001., SIS Hand Function Subscale ρ=0.58 p<0.001, SIS Mobility Scale ρ=0.23  Affected arm activity: MAL-26 AOU Scale ρ =0.58, p<0.001; MAL-26 QOM Scale ρ =0.65, p<0.001, SIS Hand Function Subscale ρ=0.61 p<0.001, SIS Mobility Scale ρ=0.41 p<0.001 |
| Waddell & Lang 2018^33^ | Outpatient rehabilitation | Actigraph wGT3X+, Pensacola, FL | 24 hrs at baseline and again post-intervention during all daily activities | Use ratio | Not reported | Not reported | MAL and use ratio were moderately associated at baseline (r=0.31, 95% CI [0.07, 0.51], p=0.01) and post-intervention (r=0.52, 95% CI [0.32, 0.68], p<0.001) |
| Wang et al., 2011^38^ | Medical centres | Not reported | All day | Not reported | Not reported | Correlations were considered low (0-0.25), fair (0.25-0.5), moderate to good (0.5-0.75), and good to excellent (>0.75) | ABILHAND and accelerometer data *r*=0.45–0.54 |
